# A method for evaluating population and infrastructure exposed to natural hazards: tests and results for two recent Tonga tsunamis

**DOI:** 10.1186/s40677-023-00235-8

**Published:** 2023-02-16

**Authors:** Bruce Enki Oscar Thomas, Jean Roger, Yanni Gunnell, Salman Ashraf

**Affiliations:** 1 Institute of Geodesy (GIS), University of Stuttgart, Stuttgart, Germany; 2 Earth Structure and Processes, GNS Science, Lower Hutt, New Zealand; 3 Université Lumière Lyon 2, CNRS UMR 5600, Bron, France; 4 Data Science and Geohazards Monitoring, GNS Science, Lower Hutt, New Zealand

**Keywords:** Kingdom of Tonga, Tsunami, Impact assessment, Population inventory, GIS, Dasymetric mapping

## Abstract

**Background:**

Coastal communities are highly exposed to ocean- and -related hazards but often lack an accurate population and infrastructure database. On January 15, 2022 and for many days thereafter, the Kingdom of Tonga was cut off from the rest of the world by a destructive tsunami associated with the Hunga Tonga Hunga Ha’apai volcanic eruption. This situation was made worse by COVID-19-related lockdowns and no precise idea of the magnitude and pattern of destruction incurred, confirming Tonga’s position as second out of 172 countries ranked by the World Risk Index 2018. The occurrence of such events in remote island communities highlights the need for (1) precisely knowing the distribution of buildings, and (2) evaluating what proportion of those would be vulnerable to a tsunami.

**Methods and Results:**

A GIS-based dasymetric mapping method, previously tested in New Caledonia for assessing and calibrating population distribution at high resolution, is improved and implemented in less than a day to jointly map population clusters and critical elevation contours based on runup scenarios, and is tested against destruction patterns independently recorded in Tonga after the two recent tsunamis of 2009 and 2022. Results show that ~ 62% of the population of Tonga lives in well-defined clusters between sea level and the 15 m elevation contour. The patterns of vulnerability thus obtained for each island of the archipelago allow exposure and potential for cumulative damage to be ranked as a function of tsunami magnitude and source area.

**Conclusions:**

By relying on low-cost tools and incomplete datasets for rapid implementation in the context of natural disasters, this approach works for all types of natural hazards, is easily transferable to other insular settings, can assist in guiding emergency rescue targets, and can help to elaborate future land-use planning priorities for disaster risk reduction purposes.

**Supplementary Information:**

The online version contains supplementary material available at 10.1186/s40677-023-00235-8.

## Introduction

### The 15 January 2022 tsunami event and its impact

**Fig. 1:**
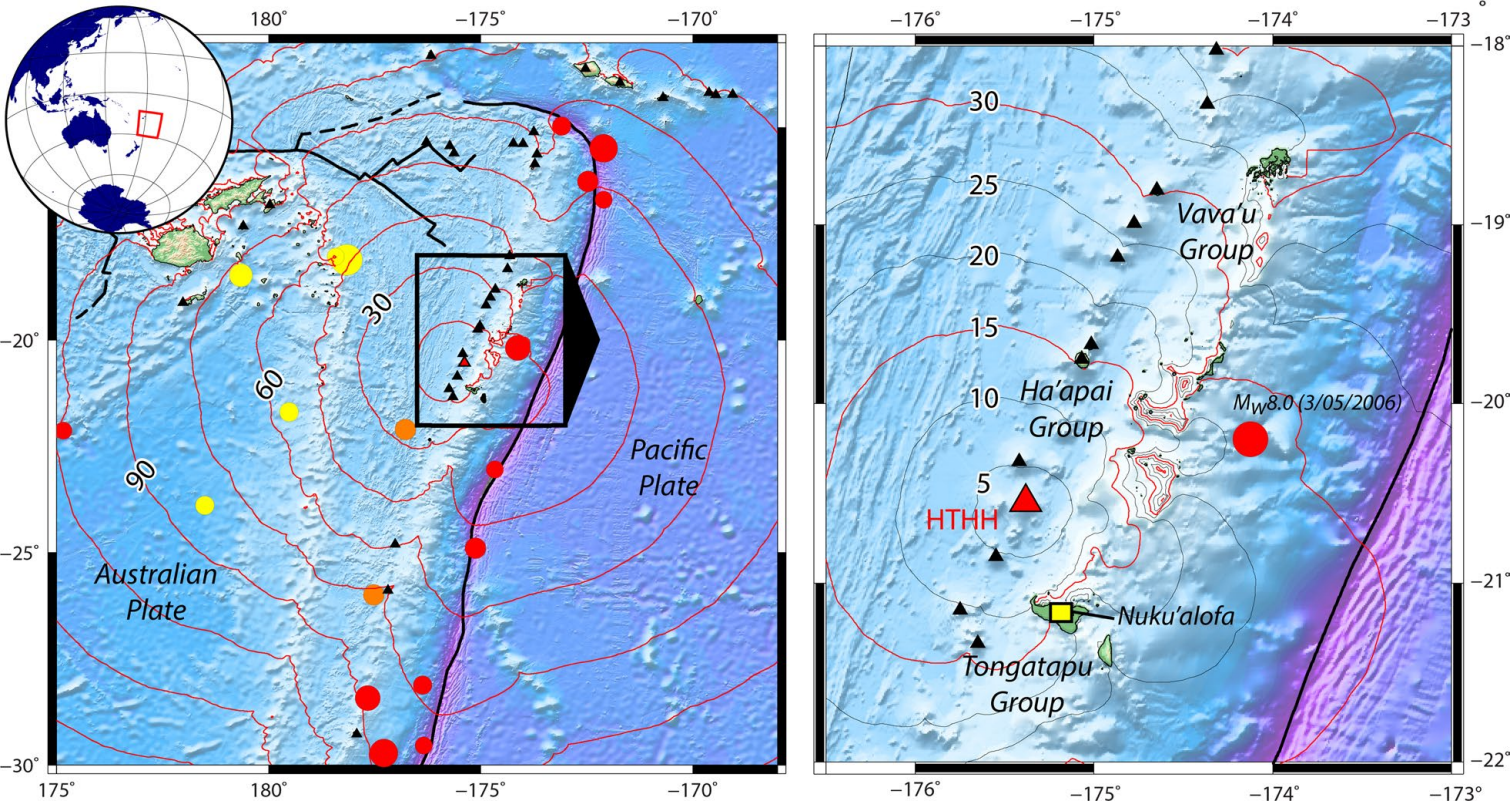
Geological hazards in Tonga. Left panel (framed by red rectangle on globe): seismotectonics around the Tonga–Kermadec subduction zone. Right panel (framed by black rectangle in left panel): the three main island groups of Tonga (Vava’u, Ha’apai and Tongatapu). Circles: epicentres of magnitude Mw > 7.5 earthquakes (circle sizes proportional to magnitude; USGS data from January 1970 to February 2022) with color as a function of focal depth: shallow (red), intermediate (orange), deep (yellow). Black lines: main tectonic features (subduction trench, spreading ridges). Black triangles: active Holocene volcanoes (https://volcano.si.edu, accessed on February 13, 2022). Red triangle: Hunga Tonga-Hunga Ha'apai (HTHH) volcano. Red and black contours: tsunami travel times from the volcano, in minutes (calculated using Mirone Software; Luis 2007). Bathymetric data from GEBCO (2014). Map generated using Generic Mapping Tools (Wessel et al. 2019)

On January 15, 2022, the violent eruption of an underwater volcano in the Southwestern Pacific Ocean (175.385° W, 20.565° S) entailed disastrous consequences on neighboring islands of the Tonga archipelago (Fig. 1). In addition to the volcanic products (mostly ash) and to the massive airborne shockwave generated by the eruption and recorded around the world (Gusman et al. 2022), the eruption was followed by a tsunami that was promptly recorded on nearby gauges and DART sensors (Gusman and Roger 2022). A Pacific-wide tsunami threat bulletin was issued, and coastal populations were evacuated on New Zealand’s north island (Hunt and Piper 2022; NZ Herald 2022), in New Caledonia (LNC 2022), and as far as Japan, where 229,000 residents were moved to higher ground (Imamura et al. 2022; Japan Times 2022).

The maximum amplitude of the tsunami wave was generally less than 1 m, but for some islands located only tens of kilometers away from the volcano, the waves were much higher and destructive, and suspected to be associated with one or several submarine landslide events (Lynett et al. 2022). Several deep-sea telecommunications cables were severed, therefore prohibiting easy communications (GFDRR 2022; Terry et al. 2022). Moreover, the general pandemic situation in a COVID-19-free island kingdom (BBC 2022; Vainikolo 2021) impeded opportunities for investigating the impact of the tsunami directly on the ground in the days following the event. Thus, the number of tsunami-impacted people and buildings remained unknown for many weeks and generated uncertainty around how to prioritize immediate international help to Tonga. The United Nations Institute for Training and Research (UNITAR) released preliminary information only two days after the event, in a satellite-derived damage assessment report showing that many buildings had been destroyed by the tsunami (OCHA 2022a). The evidence was published 20 days later by UNOSAT (2022). Based on the topographic elevation of those buildings, it appears that the tsunami reached + 15 m on Tongatapu, ‘Eua and Ha’apai islands (Government of Tonga 2022). This satellite-based analysis has so far revealed that more than 80% of Tonga was affected by the disaster, whether by thick ash fall or the tsunami (OCHA 2022a).

### Context

The Kingdom of Tonga is a southwest Pacific country composed of 172 islands of surface area above 0.005 km^2^, 45 of them inhabited by a total population of 100,651 (2016 census, TSD 2019). About 70% of this population is concentrated on the main island, Tongatapu (~ 260 km^2^), which hosts the capital city Nuku’alofa. The Tonga Statistics Department (TSD) indicates that 27% of the country’s population is poor, living monthly on less than TOP$ 970 (Tongan Pa’anga, equivalent to US$ 428 on February 13, 2022). According to World Bank assessments, ~ 3% of Tonga’s population lives in extreme poverty. This refers to people with a monthly income of less than TOP$ 3.10 (= US$ 1.37 on February 13, 2022; Fifita et al. 2018).

These islands are volcanic edifices associated with the subduction of the Pacific Plate beneath the Australian Plate. Some of the volcanoes are extinct and subsided/eroded (now mostly coral islands), but others are still active (Bryan et al. 1972). The island population is thus exposed to strong geological hazards like subduction megathrust earthquakes exceeding magnitudes of *M*_w_ 8.0, explosive volcanism, subaerial and submarine landslides originating on volcanic slopes, and tsunamis. Recent reminders of the destructive capacity of these processes include the September 9, 1946 eruption, which led to a definitive evacuation of the population from Niuafo’ou (Rogers 1981); the June 23, 1977 *M*_w_ 7.2 earthquake, which caused major damage on Tongatapu and’Eua (Campbell et al. 1977); the May 3, 2006 *M*_w_ 8.0 earthquake, which caused damages and a small tsunami in southern Tonga (Cummins et al. 2007; Heeszel et al. 2006; Tang et al. 2008); and the September 29, 2009 tsunami triggered by a *M*_w_ 8.1 and 8.0 earthquake doublet (Clark et al. 2011; Fritz et al. 2011; Lay et al. 2010). Traces of past far-field events have also been highlighted by several studies (e.g. Okal et al. 2004), including the controversial occurrence of tsunami-related boulder deposits (Frohlich et al. 2009; Lavigne et al. 2021).

Tonga is also situated along the path of devastating tropical cyclones such as Isaac (March 1982; Reardon and Oliver 1983), Waka (December 2001; Hall 2004), Ian (January 2014; Havealeta et al. 2017), and Gita (January 2018; Caritas 2018). The islands are also listed globally among the most vulnerable to sea-level rise (Magee et al. 2016; Mimura 1999).

Given its levels of exposure to the aforementioned hazards, the Kingdom of Tonga holds second place among 172 countries covered by the World Risk Index 2018 (index value of 29.42: very high; World Risk Report 2018). With the help of foreign partners, the government has accordingly been developing risk assessment and preparedness plans, including coastal development and emergency management consolidation within the framework of international disaster risk reduction guidelines (Bolton et al. 2020; Fakhruddin et al. 2019; Jayavanth et al. 2009; Sattler et al. 2020; Simpson et al. 2011). Freely available data providing population numbers and building locations are embedded in census and government reports, and these are examined below in the methodology and discussion sections. Although relatively incomplete and imprecise compared to datasets available in wealthier countries, the reliability of the information they contain can be enhanced by data cleansing and systematic cross-checking.

### Objectives of the study

We propose a simple methodology dependent on a small quantity of open-access data for performing rapid and relatively accurate assessments of the numbers of people and buildings affected by a tsunami and, by extension, by other types of natural hazards within a defined region. While showcasing its potential for two tsunamis recorded in 2009 and 2022, the results and discussion provide insights into the human and infrastructural vulnerabilities in Tonga.

## Data and methodology: implementation of a low-cost toolbox for disaster vulnerability mapping

This study follows a dasymetric approach for mapping settlements and estimating population numbers that are potentially vulnerable to natural hazards. Unlike the spatially-averaged approach commonly used for generating choropleth map units, population data is redistributed into dasymetric map units based on a combination of areal weighting and the estimated population densities. The spatial heterogeneity of variables such as building and population density is thus much more accurate than in the case of choropleth maps, in which information is uniformly averaged across map units. The motivation to use the dasymetric mapping methodology, detailed below in several steps and implemented with the open-source QGIS package, is that it is fully adapted to the context of Tonga, where open-access datasets are available for representing the distribution of population and buildings before the 2022 tsunami. The data include (i) opensource GIS layer of buildings, (ii) Google Earth images showing buildings, (iii) household information, and (iv) population numbers from the latest census. The vulnerability maps and estimates of potential victim numbers were also tested against the results of a satellite-based post-tsunami damage assessment survey of Tonga generated by UNOSAT (2022).

### Step 1: operational definition of the coastal belt

A band of terrain exposed to the hazard of interest and containing potentially vulnerable settlements needs to be delineated. Some studies focus on a loosely defined coastal belt a few kilometers wide (Andrew et al. 2019; Finkl 2004), but precise operational definitions in tropical Pacific island contexts remain scarce (Dickinson et al. 1994; Eliot et al. 2020; Nunn and Campbell 2020; Nunn and McNamara 2019). In Tonga, one previous approach defined the coastal belt based on vegetation criteria (Sykes 1981; Burley 2007). However, for assessing tsunami hazards such criteria are no substitute for run-up height as the best reference frame. In this paper, the coastal belt is defined between the highest astronomical tide limit and a user-defined critical elevation contour. The shoreline is defined using the Global Self-consistent, Hierarchical, High-resolution Geography database (GSHHG 2017). Given the small size of the islands and the highest run-ups reported (Clark et al. 2011; Fritz et al. 2011; Government of Tonga 2022), the 0–30 m elevation band covers a comprehensive range of possibilities from small to potentially large tsunamis. In this study, topographic data are extracted from the open-access Shuttle Radar Topography Mission (SRTM) global dataset (SRTM 2015), which has a horizontal resolution of 1 arc-second (~ 30 m at the equator) and a minimum vertical accuracy of 16 m with 90% confidence (Mukul et al. 2017). The vertical reference datum for the SRTM dataset is mean sea level.

### Step 2: inventory of population distribution data

**Fig. 2:**
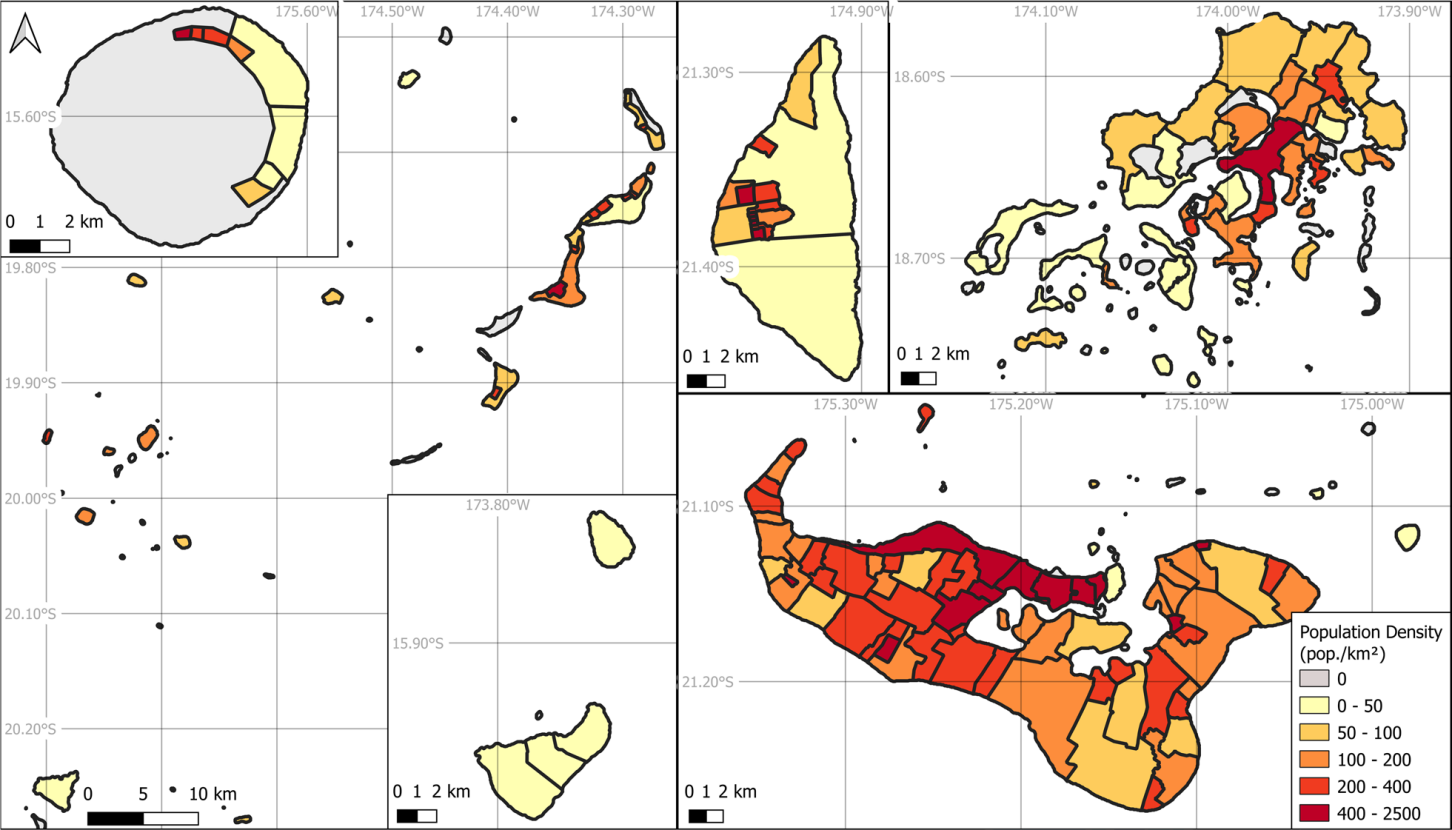
Population density (pop./km^2^) in Tonga by villages (TSD 2017)

The 2016 census provided crucial information about population distribution at four administrative levels: country, division, district, and village (levels 0 to 3) (TSD 2017, 2019; note that the 2021 census was still in its preliminary stage in the aftermath of the HTHH eruption and, therefore, not used in this study). The smaller census units are thus the 166 villages scattered across the archipelago, with their boundaries identified in a GIS layer dating from February 2018 (OCHA 2022b). The population density of each village varies considerably, as shown in Fig. 2. The largest island, Ha’atu’a (37.78 km^2^) hosts 522 inhabitants; the smallest, Fata’ulua (0.09 km^2^), 227; and the most populated, Kolofo’ou (3.62 km^2^): 8265.

### Step 3: inventory and distribution of buildings

The vector-format GIS database (OCHA 2022c) documents most of the infrastructure in the country. It was automatically extracted from satellite imagery and confirmed by ground-truth checks, some of them on the main island. The 2016 census also reports household counts per village, differentiating between private houses and institutional buildings (TSD 2019). However, an absence of data was apparent in 12 villages, most of them with reported residents (Table 1), and 16 areas were identified as containing buildings but not situated within the boundaries of a village—thus lacking population figures (Table 2).

**Table 1:** Update of the GIS layer of buildings for 11 villages using Google Earth

Village	Population in census	No. of buildings in census	No. of buildings mapped from Google Earth in the GIS layer
Feletoa	363	58	92
Hunga	178	39	90
Vaka’eitu	24	5	15
Makave	381	72	84
Matamaka	79	23	46
Mounu	1	1	2
Nuapapu	98	23	34
Okoa	261	48	68
Ovaka	97	20	31
Ta’anea	644	128	133
Utungake	285	57	70
Euakafa	6	2	7

**Table 2:** Population estimates for undefined villages

Undefined villages	No. of buildings in GIS layer	Population calculated on the basis of 5.5 individuals/household
Futu	4	22
Kao	1	6
Kelefesia	3	17
Lotuma	7	39
Mafana	1	6
Makaha’a	2	11
Mandala Resort	2	11
Mu’omu’a	1	6
Niniva	2	11
Nuku	2	11
Nuku’alofa Harbor	84	462
Oneata	4	22
Tofua	1	6
Tonumeia	2	11
Uoleva	15	83
Uonukuhihifo	1	6

### Step 4: identification and correction of errors by cross-verification between datasets

Given that some of the datasets contain gaps, it was on occasions difficult to link a specific village with a population statistic to a spatial distribution of buildings. A cross-verification between the four datasets was conducted in order to identify errors.

Table 1 shows 11 villages with reported inhabitants and buildings in the census but no buildings reported in the GIS layer. For this study, the GIS layer of buildings was manually completed and updated through detailed visual inspection of all the villages using imagery captured in 2018 and provided by Google Earth (Table 1). As a result, the number of buildings in the GIS layer does not exactly match the number given in the census but stays in the same range. To explain this difference, we assumed (i) that any new houses and infrastructures had been constructed since the 2016 census, and (ii) that our method of building delimitation differed from the one used in the census (for example, it is likely that we would define a house and garage or outhouse as two buildings, instead of one in the census). The population data were kept identical. On the basis of the Google Earth search, this study confirms that all types of infrastructure, including makeshift dwellings, were taken into account.

Table 2 focuses on the 16 areas situated outside village perimeters but with buildings reported in the GIS layer. No link can be established with an existing village or population number. In this study, these areas were termed *undefined villages* and considered as census units on the same basis as other villages. The presence of buildings, whether residential or not, in the GIS layer indicates that the population of these undefined villages needs to be included in the same way as the other villages (Table 2). For all other Tonga islands, the census indicates an average household size of 5.5 people (Scott and Browne 1989; TSD 2019). On that basis, the population of undefined villages was estimated as follows: no. of buildings × 5.5, rounded to the nearest unit.

### Step 5: delineation of built-up areas

**Fig. 3:**
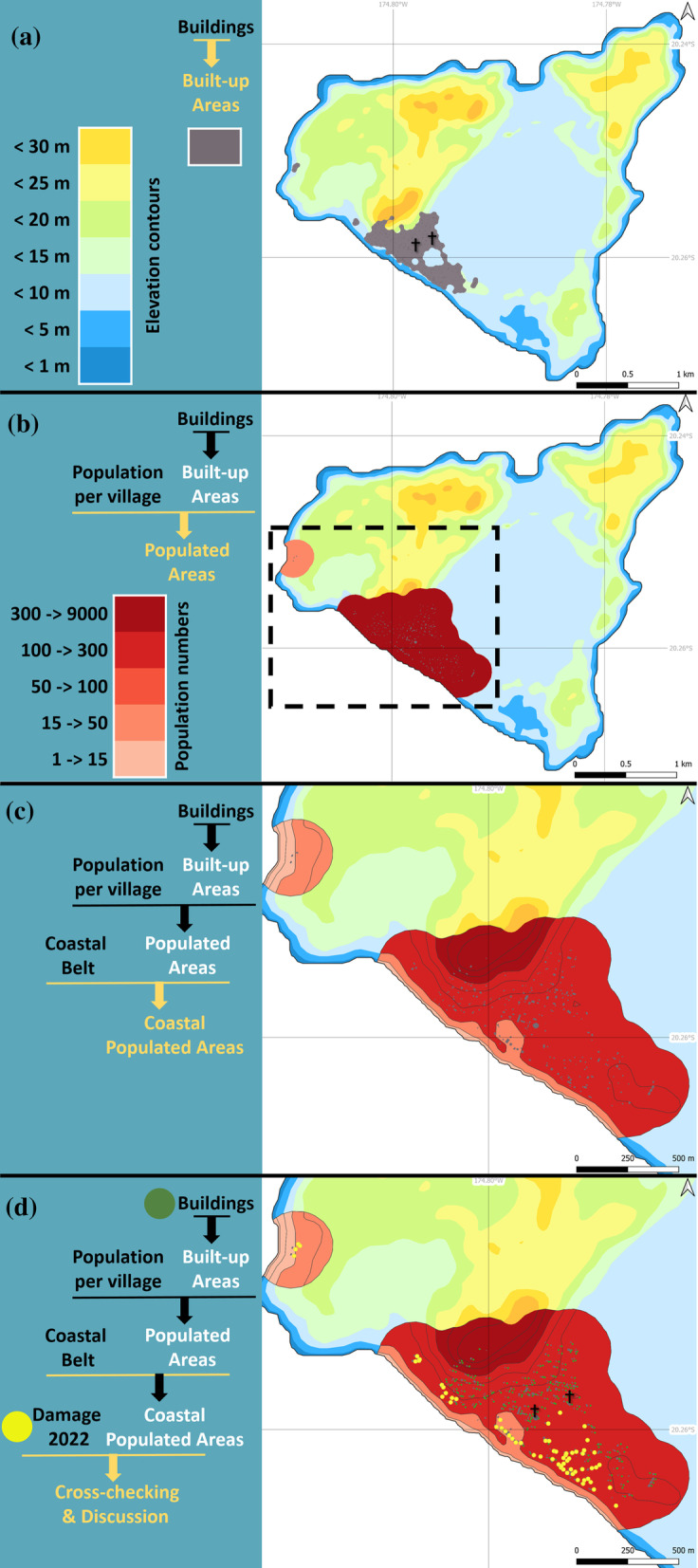
Methodology describing the implementation of a low-cost toolbox for disaster vulnerability mapping. Components of the seven steps are displayed in the right panels. **a** Steps 3, 4 and 5 use the buildings inventory to define built-up areas. **b** Steps 2, 4 and 6 use the population inventory to define populated areas. **c** Steps 1 and 7 use an operational definition of the coastal belt to outline coastal populated areas. **d** Step 8 uses damage inventory to discuss the model quality. Green dots: buildings (OCHA 2022c). Yellow dots: buildings damaged by the tsunami in 2022 (CEMS 2022; USGS HDDS 2022). Black crosses: churches (churches are the only non-residential buildings described in the existing GIS layer: fire stations, police stations, schools and other public buildings are absent, thereby emphasizing the lacunar character of the information available)

The GIS layer of built-up areas was crafted directly from the GIS layer of buildings while excluding road networks. A process aimed at cutting away spaces between buildings was implemented while maintaining a 50 m buffer area around the buildings, and was followed by merging the polygons obtained. A further 30 m band was shaved off each resulting polygon, thus leaving a 20 m perimeter of land around each building or group of closely spaced buildings (typically < 100 m apart; Fig. 3a). These values are consistent with methods previously used (Loriot and Di Salvo 2008), take account of the density and disparity of buildings in Tonga, and are compatible with the outdoor lifestyle of Tonga’s inhabitants, who typically spend time outdoors within a 20 m radius of their residence (Bolton et al. 2020; Jin et al. 2014).

### Step 6: delineation of populated areas

This component of the dasymetric mapping approach involves defining populated areas, i.e., built-up areas where the population of the census unit is effectively concentrated (rather than spatially averaged across the census unit, as would be shown in choropleth maps). Here, populated areas were generated as a GIS vector layer defined by the outlines of the built-up areas (see Step 5) and coupled with the census population values. Note that maps from the TSD were delivered after the 2022 eruption to locate ‘populated places’ (OCHA 2022d), which is a notion similar to the populated areas we are defining here. However, we performed compatibility tests that show the data are incomplete and do not match the building data (for example, to the west of Fua’amotu International Airport, Fua’amotu village is not indicated). This study consequently ignored the populated places given in OCHA (2022d) and defined the populated areas using the built-up areas obtained at Step 5.

The boundaries of the populated areas are defined by a 200 m spatial aggregation algorithm applied to the built-up areas generated at Step 5 to take into account building dispersal, then a 50 m erosion buffer is applied to achieve a tighter geographic fit to the buildings (i.e., each building or cluster of buildings is surrounded by a uniform band of terrain 150 m wide; Thomas et al. 2021). The intersection of populated areas with census unit boundaries (in Tonga, census units are called villages) allows the population of the village to be directly associated with the corresponding unit(s) in the populated areas layer. Depending on the distribution of buildings, a village can be composed of more than one populated area (see Fig. 3b).

### Step 7: delineation of coastal populated areas

The last task involves intersecting the populated areas with the coastal band defined at Step 1, thereby defining ‘coastal populated areas’, i.e., areas lying within reach of a given tsunami run-up magnitude. This process is repeated for each elevation band up to + 30 m by increments of 5 m. The population number is then allocated to these coastal populated areas proportionally to polygon area, assuming thus a uniform population density within populated areas (Fig. 3c).

‘Coastal populated areas’ (step 7) and ‘populated areas’ (step 6) are thus directly generated by reference to the ‘built-up areas’ (step 5), which are themselves upscaled from the ‘buildings’ layer (step 3). The dasymetric mapping methodology thus operates at an aggregated level rather than at the basic building footprint level because it allows buildings potentially missing from the data layer (e.g., new, or unreported) to be included (thereby erring on the side of caution in the risk assessment exercise). It also allows areas adjoining buildings (within the previously defined 150 m buffer belt) to be included as daily living space because life in Pacific island cultures includes a lot of time spent outdoors.

### Step 8: model quality assessment from independent damage data

The damage assessment data were retrieved from the EU’s Copernicus Emergency Management Service (CEMS) and the United Nations Satellite Centre (UNOSAT), hosted by UNITAR. The data consist of different vector layers as ESRI shape files, Google Earth KML and GeoJSON formats (CEMS 2022; USGS HDDS 2022), prepared using visual interpretation of pre- and post-event high-resolution satellite images shared by the USGS Hazards Data Distribution System (HDDS) portal (public satellite sources). The visual inspection of images highlighted the damaged buildings, tsunami-related shoreline changes, and the assessment of flooding extent (Fig. 3d).

## Results

This rapid, low-cost methodology for assessing tsunami impacts on humans and infrastructure delivers a number of dasymetric vulnerability maps. All the open-access shapefiles of the populated areas are accessible in greater detail in the Additional file 1. Here we present some result highlights for later discussion.

**Fig. 4:**
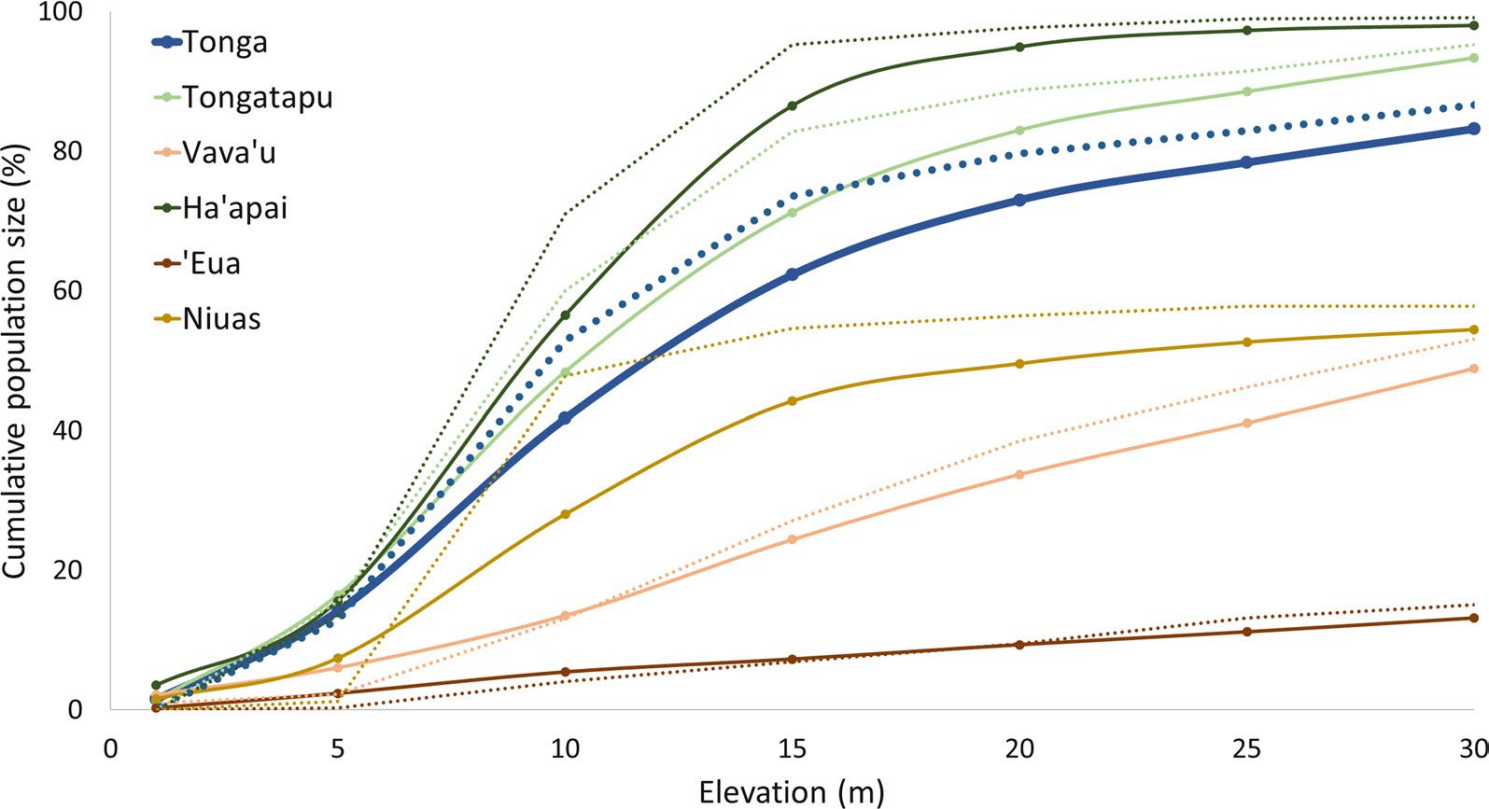
Cumulative distribution of population and buildings for all Tonga and for each of the 5 divisions (group of islands), paired by color as a function of elevation. Full lines: population distribution. Dotted lines: buildings distribution

For each user-defined coastal elevation band and for each village, a precise estimation of population and building numbers are given in Additional file 2. Those figures serve as a quick reference tool for identifying and locating where the highest disaster management challenges occur in the landscape. An extract of these data is presented in Table 3 and the full dataset for Tonga is plotted in Fig. 4. Strong contrasts in population distribution between each of the five island groups are highlighted.

**Table 3:** Example of number of inhabitants and buildings by village, estimated for different coastal belt widths

Village name	< 1033 m (highest elevation)		< 25 m elevation				< 5 m elevation			
	Population	Buildings	Population		Buildings		Population		Buildings	
	(No.)	(No.)	(No.)	(%)	(No.)	(%)	(No.)	(%)	(No.)	(%)
Pangaimotu	657	204	386	59	164	80	47	7	3	1
‘Utulei	116	48	52	45	30	63	19	16	0	0
Nga’unoho	181	69	149	82	66	96	36	20	23	33
‘Utungake	285	73	203	71	70	96	111	39	46	63
Tapana	3	6	3	100	6	100	1	33	1	17

**Fig. 5:**
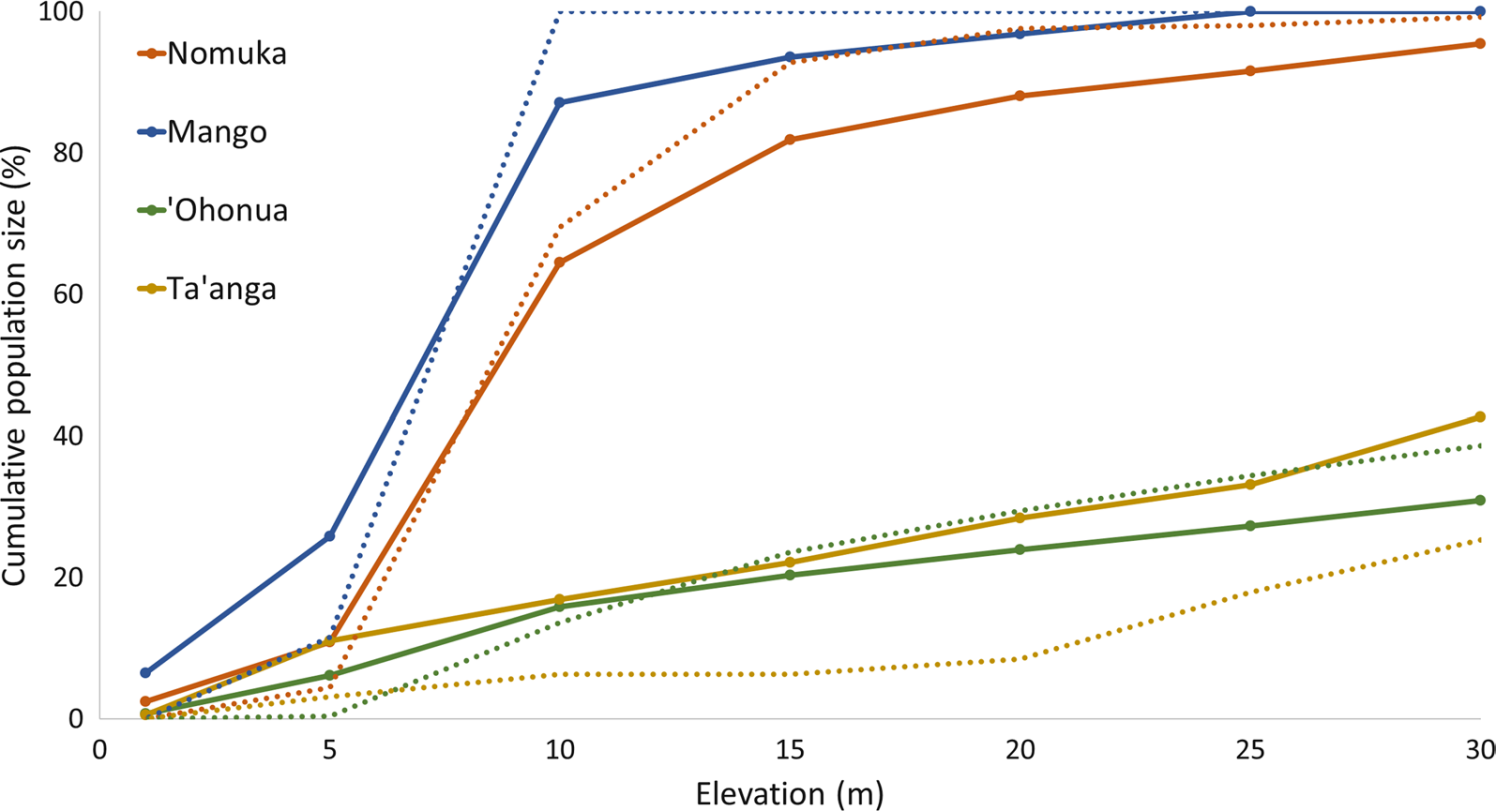
Cumulative distribution of population and buildings in four selected villages damaged by the tsunami in 2022. Full lines: population distribution. Dotted lines: buildings distribution. Nomuka and Mango belong to the Ha’apai island group. ‘Ohonua and Ta’anga are in ‘Eua group

The Ha’apai group is composed of 68, mostly low-lying islands with more than 50% of the global Tongan population and more than 70% of the buildings located below the 10 m elevation contour. ‘Eua is hilly, with peaks above 300 m and more than 85% of the population living above 30 m. Both island groups were struck by the 2022 tsunami, with several buildings damaged. Figure 5 provides a close-up of some of the villages impacted by the tsunami. In all villages, a jump in population and building numbers is observed between 5 and 10 m, revealing a concentration of human settlements below the 10 m elevation contour and thus greater vulnerability to coastal hazards.

**Fig. 6:**
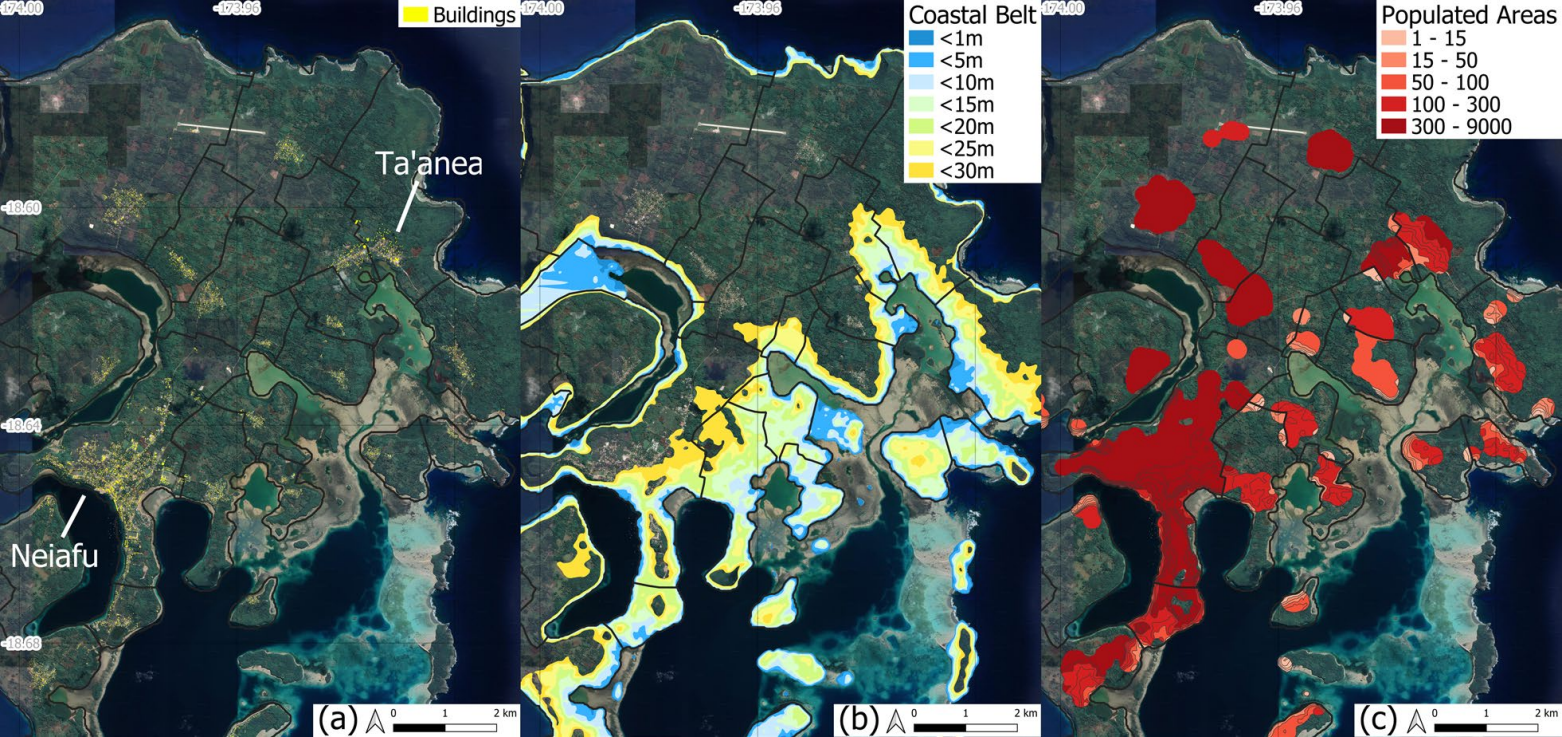
Example of dasymetric maps produced for locating populated areas in Tonga. **a** Buildings layer on Google Earth imagery background. **b** Coastal belts below 30 m elevation. **c** Coastal populated areas

An efficient way of understanding those datasets consists in mapping the entire population on each island, and then focusing on the populated areas that pinpoint in each village where the population actually lives (Thomas et al. 2021). The result presented here focus on the Vava’u group of islands, around the village of Ta’anea, in order to illustrate the methodology (Fig. 6). The GIS layers are available in the Additional file 1 in order to zoom in.

This study estimates overall that 83% of the population of Tonga lives within the 0–30 m elevation band, and 42% lives below 10 m. Likewise, 87% of buildings occur below the 30 m, and 53% below the 10 m contour. At least 14% of the population lives below 5 m. Finally, the graphs clearly highlight the large disparity in population and building densities between two villages situated in contrasting topographic settings.

## Discussion

Here we test the predictive power of the methodology based on two real tsunamis in Tonga: the January 15, 2022 and September 29, 2009 events. We also show the relevance of this methodology for other types of natural hazards such as storms, volcanic eruptions, and earthquakes.

### Case study #1: 2022 tsunami

Satellite imagery has revealed considerable damage at locations on Tongatapu, ‘Eua and Ha’apai islands, suggesting that run-ups reached the 15 m contour (Government of Tonga 2022; UNOSAT 2022). Figure 4 indicated that 62% of the population (n = 62,677) lives below the 15 m contour in all of Tonga (61% when restricting to Tongatapu, ‘Eua and Ha’apai islands). The CARE (2022) report recorded 84,776 affected people from the tsunami and volcanic fallout combined. Our map-derived estimations indicate that 74% of buildings in Tonga are located in a coastal tsunami hazard area below 15 m, meaning all those buildings may have suffered from tsunami run-ups in 2022. The additional impact of thick ash fall causing roofs to collapse tallies with reports claiming that this double disaster affected 80% of Tonga (OCHA 2022a) and caused almost 100% damage to buildings (GFDRR 2022). Here we gather information restricted to tsunami damage and discuss links with the results obtained through the methodology.

UNOSAT (2022) provides an overview of the damage to buildings for several districts through satellite imagery analysis. An example of this information is given in Table 4, with direct links to the number of buildings, populated areas and inhabitants for each district analyzed. As shown, the information supplied is imprecise but can be advantageously refined by using the dasymetric maps produced in this study for obtaining a rapid assessment of the situation and a first-order, reasonably accurate estimation of vulnerable buildings and number of residents in population clusters. As explained in Fig. 5, ‘Eua and Ha’apai shows sharp contrasts in topography, but the tsunami had similar impacts on structures standing below the 15 m contour on both islands.

**Table 4:** Comparison of damages to buildings between UNOSAT (2022) data and results from this study

Information provided by UNOSAT (2022)			Information gained from this study		
Island	District	Buildings damaged	No. of populated area units	Existing buildings	Estimated no. of inhabitants
‘Eua	Fo’ou	1	13	776	2150
	Prope	139	21	1058	2795
Ha’apai	Lulunga	11	7	325	923
	Mu’omu’a	145	4	305	432
	‘Uiha	4	5	407	695

UNOSAT Maps (2022) provides an atlas of disaster-related damages on Tonga based on satellite imagery. The assessment focuses on a selection of villages highly affected by the tsunami, with a confirmed number of damaged buildings. The methodology implemented here shows an improvement in detecting the number of potential buildings impacted, while also linking the geographic information directly to an estimated maximum number of vulnerable inhabitants (Table 5).

**Table 5:** Comparison of damages to buildings in specific populated areas

Information provided by UNOSAT Maps (2022)		Information gained from this study			
Location	No. of buildings damaged	Populated area	Elevation (m)	No. of buildings	No. of inhabitants
Tungua	11	Tungua	< 10	82	178
Fonoifua (Mu’omu’a)	30	Fonoifua	< 10	30	25
‘Atata	72	‘Atata	< 10	89	122
‘Eua	48	‘Ohonua (Prope)	< 15	118	254
	6	Ta’anga (Prope)	< 15	6	42
	1	Futu (Fo’ou)	< 20	7	23
Mango (Mu’omu’a)	26	Mango	< 10	26	27
Nomuka (Mu’omu’a)	61	Nomuka1	< 10	184	227
	4	Nomuka2	< 15	4	28

Our estimation of buildings damaged is often close to, although occasionally much higher, than the imagery-based numbers reported by the UNOSAT survey. Compared to the existing dataset of buildings per village, this overestimation clearly highlights an improvement in the results (Table 6). A comparison coefficient *c*, defined below, is calculated to quantify the gain in accuracy when estimating vulnerable buildings using the three datasets: (i) the number of buildings in the village, *bv*, based on the TSD (2019) data; (ii) the number of buildings in the populated area, *bpa*, identified in this study; and (iii) the number of effectively damaged buildings, *bd*, using the UNOSAT Maps (2022).

$$c = \frac{{\left( {bv - bpa} \right)}}{{\left( {bv - bd} \right)}}$$

Coefficient *c* ranges between 0 and 1. The closer *c* gets to 1, the closer the estimation from this study approximates the actual damage estimated by UNOSAT. As *c* approaches 0, the estimation obtained stays close to the TSD data, with little improvement. Among the examples compiled in Table 6, improvement in the precise knowledge of vulnerable buildings is gained for 4 locations, where *c* > 0.85 (‘Ohonua, Ta’anga, Futu, and Nomuka2). Tungua, ‘Atata and Nomuka1 perform less well, perhaps because our approach only considers the run-up distance of the tsunami, i.e., the elevation range of the impact, and not the form of tsunami propagation or disparities in the intrinsic mechanical strength of the buildings exposed to it (our estimation is based on the assumption that all the buildings in the relevant coastal band are damaged). Fonoifua and Mango were completely destroyed by the tsunami. This is confirmed by numbers from the TSD dataset as well as by this study’s dataset, and provides in itself a validation of the methodology. Furthermore, any overprediction of building damage in this study may advantageously compensate for likely errors in the definition of the coastal belts when based on SRTM elevation data, which have generated cases of underprediction in other studies (Kulp and Strauss 2016).

**Table 6:** Estimation of damages to buildings in specific populated areas: a comparison between three datasets

Location	No. of buildings			Coefficient *c*
	Damaged *bd*^a^	In the populated area *bpa*^b^	In the village *bv*^c^	
Tungua	11	82	83	0.01
Fonoifua	30	30	30	1
‘Atata	72	89	104	0.47
‘Ohonua	48	118	500	0.85
Ta’anga	6	6	95	1
Futu	1	7	99	0.94
Mango	26	26	26	1
Nomuka1	61	184	249	0.41
Nomuka2	4	4	249	1

^a^From UNOSAT Maps (2022); ^b^this study; ^c^from TSD (2019)

**Fig. 7:**
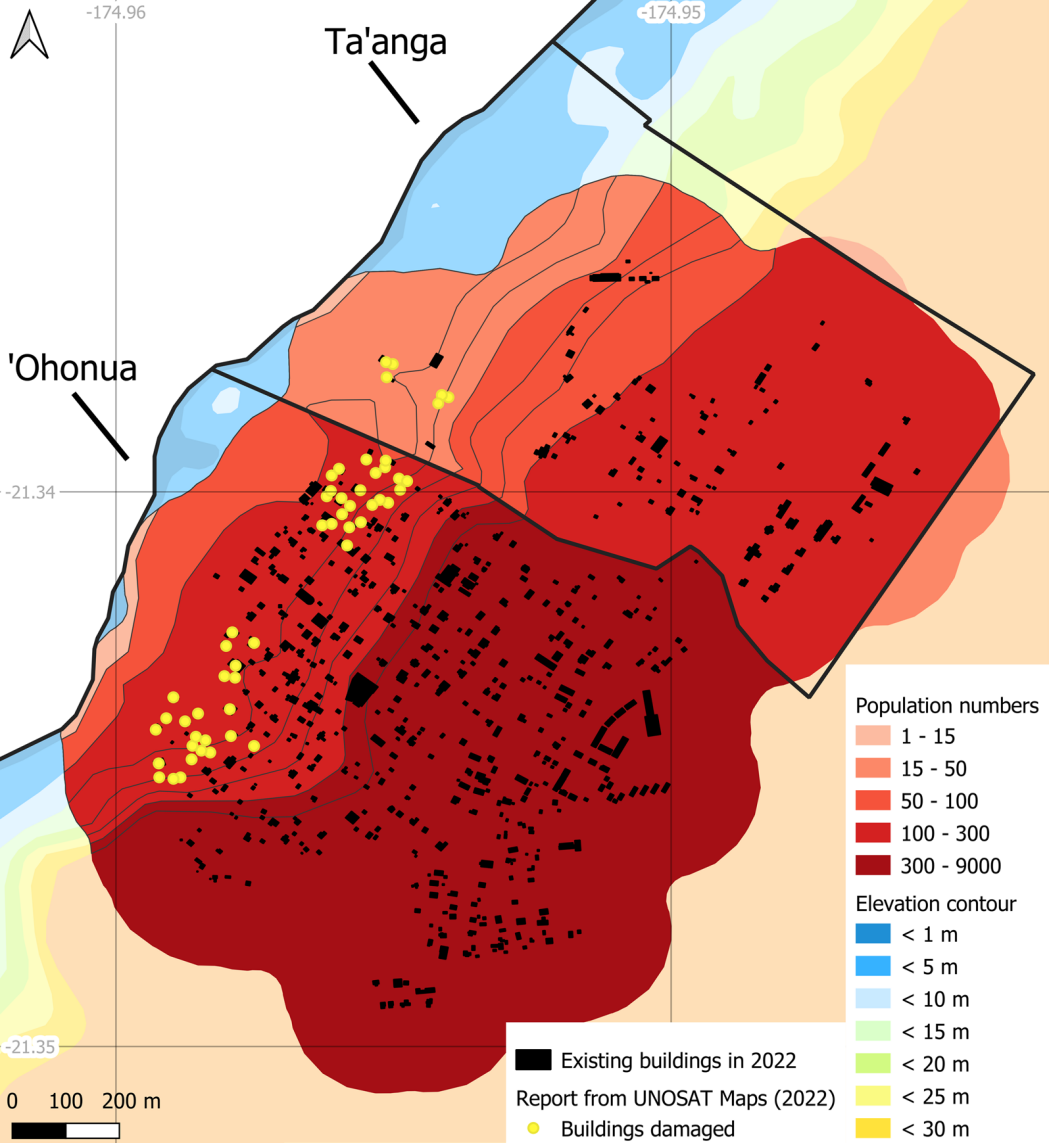
Detailed map of individual buildings damaged by the 2022 tsunami on ‘Eua island. Gray polygons: buildings included in this study (OCHA 2022c). Yellow dots: buildings confirmed damaged by UNOSAT Maps (2022)

**Fig. 8:**
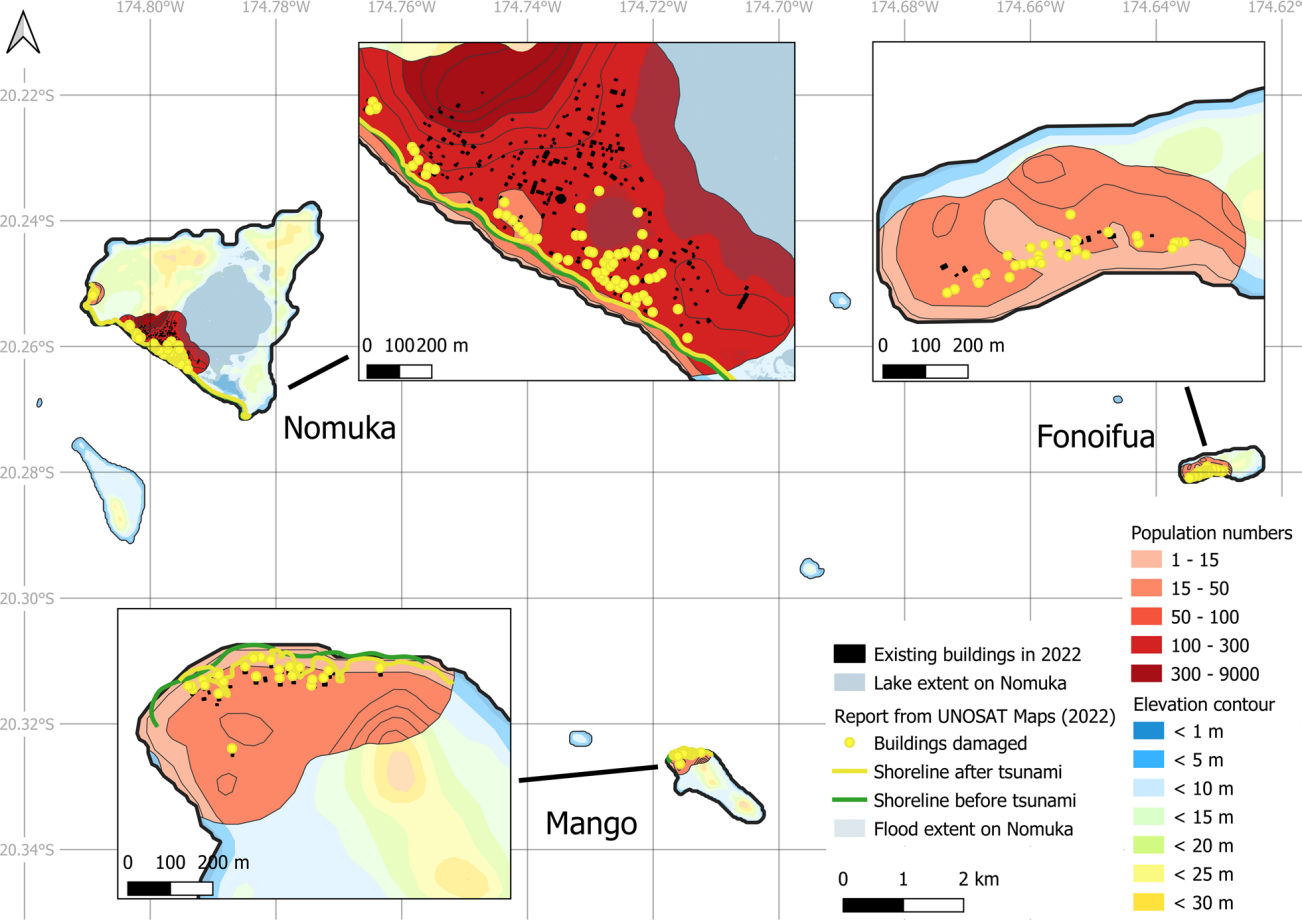
Overview of buildings damaged by the 2022 tsunami on Nomuka, Mango and Fonoifua islands. Gray polygons: buildings included in this study (OCHA 2022c). Yellow dots: buildings confirmed damaged by UNOSAT Maps (2022). Green line: shoreline before the tsunami (GSHHG 2017). Yellow line: shoreline retreat after the tsunami by UNOSAT Maps (2022)

The good match between the UNOSAT data and our mapping results presented in Tables 5 and 6 highlights the value of the methodology presented here. Geographic precision is additionally provided by the GIS maps of populated areas, here shown for ‘Eua in Fig. 7, and for the three islands of the Ha’apai group in Fig. 8, which were severely affected by the 2022 tsunami in all of Tonga. Entire villages on Mango and Fonoifua were swept away, 13 houses were flooded between the coast and the lake in Nomuka, and the shoreline retreated by up to 10 m on Nomuka and 30 m on Mango (CEMS 2022; GFDRR 2022; Pleasance 2022; UNOSAT Maps 2022).

In the context of a future tsunami event, maps such as Figs. 7 and 8 can be used to precisely locate in each village how many inhabitants were potentially vulnerable to that particular hazard and could have been affected by its estimated magnitude. Such prior knowledge can serve as an important decision-making tool. For example, by coupling the population cluster maps with tsunami hazard scenarios, this would help to focus attention on villages most exposed to a given tsunami type and propagation pattern. It would also help to calibrate the logistics of rescue operations (e.g., quantities of freshwater, first-aid equipment and food to be delivered), including passenger capacity on vessels or aircraft for the temporary relocation of disaster victims. Despite more abundant first-response and healthcare staff and facilities on the main island, residents on its west coast were nonetheless highly impacted by the 2022 tsunami. The maps and tables showcased in this study also highlight the value of precise and regularly updated census data in geographically isolated islands, particularly given that urban growth and population migration between islands occur continuously and are unlikely to decline in the near future (Lolohea 2016). For example, more than 40% of the population has moved away from the Ha’apai island group since 2011, mostly settling on Tongatapu (GFDRR 2022).

### Case study #2: 2009 tsunami

The second dasymetric mapping test case is the tsunami triggered in Samoa by the September 2009 earthquakes, claiming 9 lives on Niuatoputapu and Tahafi islands, both situated in the northeast of Tonga (Lay et al. 2010; World Bank 2022).

On Tahafi, run-ups reached 15 to 22 m on the southwestern side, damaging fishing boats and one house (Clark et al. 2011; Fritz et al. 2011; Okal et al. 2010; Wilson et al. 2009). Despite these high values, Tahafi is a steep-sided island with currently 31 inhabitants and 38 buildings counted, all situated above the 30 m contour. On Niuatoputapu, the villages of Falehau, Vaipoa and Hihifo are situated on the northwest shore, where run-ups reached the 5 m contour (Clark et al. 2011; Wilson et al. 2009). Several reports indicate around 135 to 145 buildings damaged out of a reported total of 225 to 228, mostly in the main village (Hihifo) and all occurring below the 5 m contour (Fritz et al. 2011; Government of Tonga 2009a, b; WHO 2009; World Bank 2022).

**Fig. 9:**
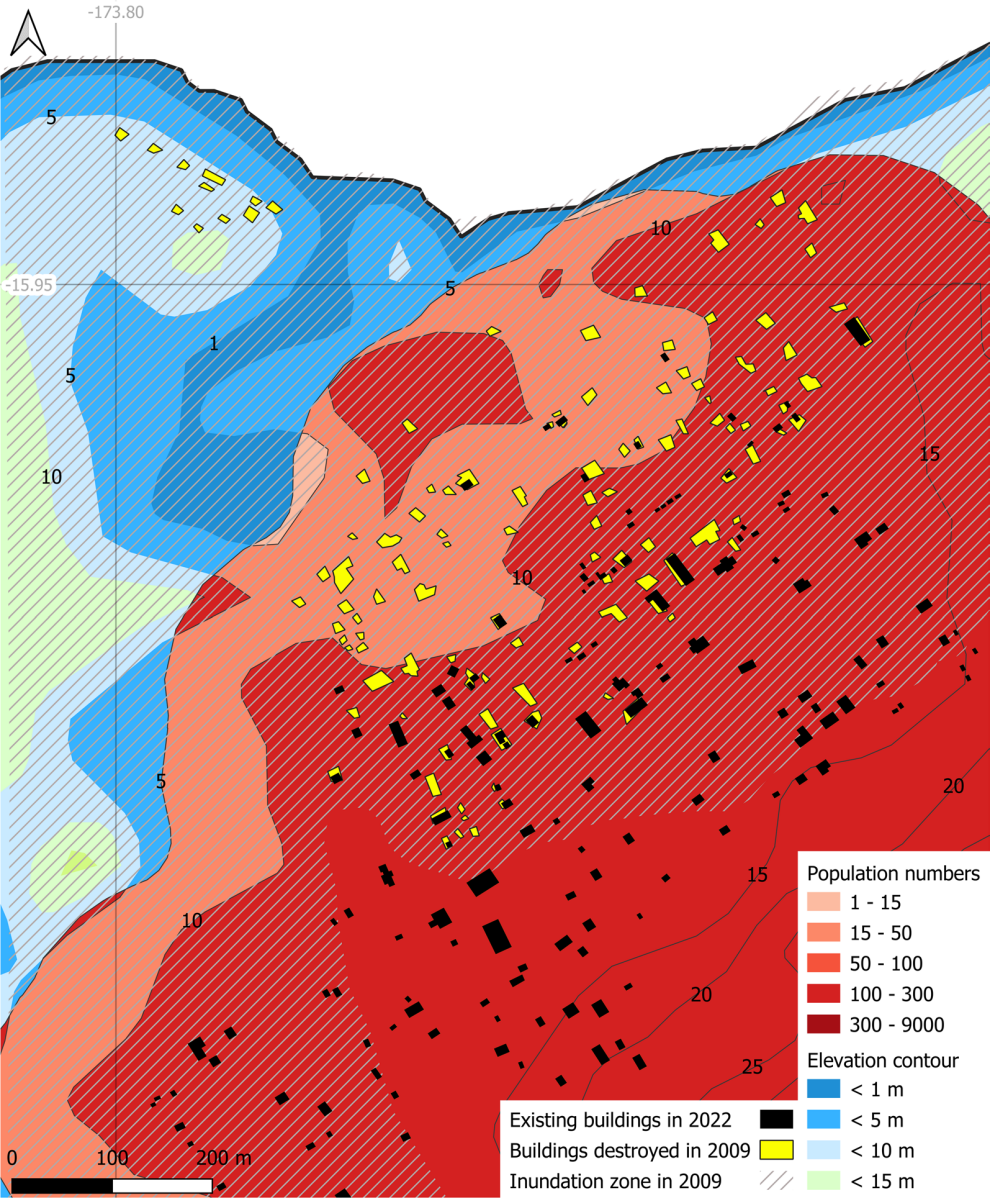
Hihifo village layout with overlay of destroyed buildings during the 2009 tsunami. Black polygons: buildings included in this study (OCHA 2022c). Yellow polygons: buildings damaged during the 2009 tsunami (Clark et al. 2011)

Using the 2016 census data, Fig. 9 displays an overlay of Hihifo village on the post-tsunami survey of building locations in 2009 (Clark et al. 2011). Only 7 buildings below the 5 m contour were spared among the 44 existing before the 2009 tsunami. Whereas 38 inhabitants are currently recorded as residing below the 5 m contour, population estimates at the time were closer to 242. These figures highlight the magnitude (57%) of post-tsunami emigration, not just in temporary buildings in Falehau (Clark et al. 2011; WHO 2009), but by a long-term decision to definitively relocate housing away from areas exposed to coastal hazards—generally to higher elevations. Niuatoputapu island nonetheless appears to benefit from the coral reef surrounding the island, which protected the villages from excessive run-up magnitudes on its northwest coast (Fritz et al. 2011). Although not universally verified, tsunami amplitudes can be mitigated by the presence of healthy coral reefs (Fernando et al. 2005; Ferrario et al. 2014; Hardy and Young 1996; Harris et al. 2018; Karim and Nandasena 2022; Kunkel et al. 2006; Monismith et al. 2015; Roger et al. 2014). This unique ecological asset should be integrated in future risk management studies on Tonga.

Higher run-ups occurred at the southern tip of Niuatoputapu along a near-shore fringing reef, with observed flow depths of up to 6 m (Wilson et al. 2009). This is consistent with the damage sustained by the airstrip, which is situated below 10 m and was partially inundated at the southern end of the runway (Clark et al. 2011). This is the only air connection to other islands in Tonga and highlights the dangers of placing communications infrastructures at low elevations. The only undamaged public building on the entire island was the high school, which stands above the 5 m contour, emphasizing here also the critical importance of choosing elevated ground for primary infrastructure whenever possible. Today, the spatial distribution of population densities and the spatial distribution of buildings appears to indicate that the hospital (completely destroyed in 2009), the primary schools, and the churches have been rebuilt above the 10 m contour. This is confirmed by several reports also indicating that water supplies, the police station, and 73 houses were built out of cyclone-resistant material and on safer and higher ground, testimony to a growing awareness of natural hazards in land-use planning in Tonga since 2009 (Government of Tonga 2009a; World Bank 2022).

### Application to other natural hazards contexts

In the aftermath of the 2009 and 2022 tsunamis, the foremost concern was to relocate residents, to clean up, and to stay on alert during the cyclone season. An accumulation of hazards hitting Tonga would significantly damage the infrastructure of the islands and strongly increase population vulnerability. The impact of the 2022 tsunami, for example, was compounded by the ash fall from the eruption, with damaging consequences beyond the tsunami-exposed coastal belt. Adding climate-change-related impacts, Tonga is thus exposed to a cumulative litany of disasters, highlighting the urgent need for disaster management strategies (Ammann 2013; GFDRR 2022; UNISDR 2005, 2009).

Although focused on coastal inundation hazard on the basis of an elevation/run-up criterion, with necessary adjustments of parameters and suitable information sources, the methodology presented in this study can address any type of natural hazard. As shown by the latest eruption of Hunga Tonga-Hunga Ha’apai, ash fall, for example, can destroy buildings even kilometers away from the eruption site. By overlapping tracking models of volcanic ash clouds with the mapping toolkit of populated areas provided here (Carn et al. 2009; Filizzola et al 2007; Searcy et al. 1998; Webley and Mastin 2009), the identification of vulnerable inhabitants can be easily assessed. Moreover, many hazardous volcanic sites, like Italy around Mt. Vesuvius (Gugg 2022), Hawai’i on Kīlauea (Meredith et al. 2022) and Java around Mt. Merapi (Garcia-Fry et al. 2022) are densely inhabited and require regular updates of populated areas. For example, when the Niuafo’ou volcano erupted in September 1946, lava flows and ash clouds destroyed infrastructures and vegetation all over the island (Rogers 1981; Taylor 1991). The entire population (1300 inhabitants) was relocated permanently on other islands. The population data from the last census show that 517 inhabitants returned permanently to the island among 8 different villages despite the volcano still being highly active (more than 10 eruptions since 1814; Taylor 1991). With 247 buildings scattered along the east coast of the island, the population is still highly vulnerable to volcanic hazards, and getting to know their precise location is a necessary feature for risk management in case of an eruption.

By its geographic position, Tonga is also exposed to strong earthquakes, such as the previously mentioned *M*_w_ 7.2 event in June 1977 causing serious damage on ‘Eua and Tongatapu islands. A similar event today would directly impact ~ 80,000 inhabitants and could damage up to 33,268 service facilities and houses. Thus, a complementary feature to the dasymetric mapping toolkit would be an updated GIS of Tonga, with all essential infrastructures explicitly positioned: civil security centers, hospitals, schools, food and freshwater supply centers. The building data could be enriched by an indication of their function in order to differentiate between residential areas and workplaces (which do not share the same population balance during day and night). A simple search on Google Maps and Maps.me confirmed how currently difficult (often impossible) it is to find accurate data about healthcare facilities on islands in Tonga. Thus, an open-source GIS stands out as an essential tool for risk management. Similar issues arise in the context of a cyclonic event such as the strongest and dramatic category-5 cyclone (Ian) of January 2014 (Havealeta et al. 2017; Johnston 2015). Damages across the Ha’apai island group were considerable, with 18 villages affected, 1094 buildings destroyed, and 2335 people relocated (Government of Tonga 2014). This study documents 6125 currently vulnerable residents on Ha’apai, and indicates that along the path taken by the cyclone Ian, 2202 inhabitants are now located among 1158 buildings. Finally, reports indicate that 17 schools were destroyed, impacting 1293 children (Government of Tonga 2014), while at the same time many infrastructures already meeting cyclone-resistant building design were saved (World Bank 2022). These examples further highlight the urgent need for a full GIS database capable of specifying precise building functions.

## Conclusion

This study focused on a rapid, low-cost approach for locating and quantifying vulnerable residents and infrastructures as precisely as possible. It involves aggregating open-access datasets, cross-checking for consistency, and generating dasymetric population maps using an open-access GIS package. Its application to the population of Tonga, which is scattered over 45 islands and lives mostly in coastal areas (~ 62% of inhabitants reside below 15 m elevation), was tested on a coastal belt defined by the limits of the highest tsunami run-up (+ 30 m) observed in the built-up areas of Tonga after the tsunami of January 15, 2022. High concentrations of built infrastructure in the tsunami exposure zone (~ 74% below the 15 m elevation contour) illustrate the high level of vulnerability of Tonga to ocean-related hazards. The mapping approach highlights the large disparity in population and building densities from one island to another, with villages positioned in a diversity of topographic settings. These results improve existing datasets from the Tonga Statistics Department by providing more accurate geographic limits for population and buildings through the coastal populated areas. In addition to documenting an entire population of scattered islands from the angle of its exposure to tsunamis (in particular the 2009 and 2022 tsunamis), we developed a dasymetric population mapping methodology—achieved in a short time span (less than two days after the 2022 tsunami) and using a small number of open-access datasets—to obtain a maximum number of vulnerable residents and buildings potentially affected by coastal hazards. Using the population data of 2016 (the only reliable dataset at the time of writing), this paper has aimed to provide a snapshot of population distribution and possible rapid decision-making actions that could have been taken following the 2022 tsunami event. By estimating a fraction of vulnerable population per populated area and affording precise visualization tools, this mapping-focused methodology is likely to appeal to a number of academic and operational stakeholders for its transferability to coastal zones more generally, and particularly to insular settings where first responders and risk management organizations need to acquire and analyze complex but reliable primary datasets. If enhanced by (1) regular updates of annual census data, by (2) monitoring of inter-island migratory movements, by (3) tracking patterns of relocation and reconstruction after disastrous events, and by (4) analyzing vulnerability patterns on remote islands with few communication links, the primary datasets and the methodology presented here can gain in accuracy, thereby assisting in planning increasingly precise and proportionate emergency rescue targets in the future.

## Supplementary Information

**Additional file 1.** Shapefiles of the populated areas in Tong, all details are provided in the associated Word document (Supplementary Material Dataset PA.docx).

**Additional file 2.** Excel file providing a precise estimation of population and building numbers for each user-defined coastal elevation band and for each village.

## Data Availability

All data generated and analyzed during this study are included in this published article and its supplementary information files.

## Abbreviations

CEMS: Copernicus Emergency Management Service
HTHH: Hunga Tonga-Hunga Ha'apai
SRTM: Shuttle Radar Topography Mission
TSD: Tonga Statistics Department
UNITAR: United Nations Institute for Training and Research
UNOSAT: United Nations Satellite Centre
USGS HDDS: United States Geological Survey Hazards Data Distribution System

## References

[CR1] Ammann WJ (2013) Disaster risk reduction. In: Bobrowsky PT (ed) Encyclopedia of natural hazards, encyclopedia of Earth sciences series. Springer, Dordrecht, pp 170–175. 10.1007/978-1-4020-4399-4_92

[CR2] Andrew NL, Bright P, de la Rua L, Teoh SJ, Vickers M (2019). Coastal proximity of populations in 22 Pacific Island countries and territories. PLoS ONE.

[CR3] BBC (2022) Tonga: how aid deliveries try to avoid bringing in Covid. BBC News Online. https://www.bbc.com/news/60066470. Accessed 2 Feb 2022

[CR4] Bolton A, Ashworth M, Akolo Fau S, Folau Fusi SK, Williamson J, Ofanoa R (2020). Strengthening adaptation planning and action to climate-related health impacts in Pacific islands countries: Tonga. Glob J Health Sci.

[CR5] Bryan WB, Stice GD, Ewart A (1972). Geology, petrography, and geochemistry of the volcanic islands of Tonga. J Geophys Res.

[CR6] Burley DV (2007) In search of Lapita and Polynesian Plainware settlements in Vava’u, Kingdom of Tonga. Oceanic explorations: Lapita and Western Pacific settlement, pp 187–198. https://www.jstor.org/stable/j.ctt24h9sg.13

[CR7] Campbell MD, McKay GR, Williams RL (1977) The Tonga earthquake of 23 June, 1977. Some initial observations. Bull N Z Soc Earthq Eng 10(4):208–218

[CR8] CARE (2022) Emergency situation report—Tonga Volcano and Tsunami Response. CARE International. https://reliefweb.int/report/tonga/emergency-situation-report-tonga-volcano-and-tsunami-response-28-february-2022. Accessed 29 March 2022

[CR9] Caritas (2018) Being prepared helps Tonga recover from cyclone Gita. https://www.caritas.org/2018/03/being-prepared-helps-tonga-recover-from-cyclone-gita/. Accessed 13 Feb 2022

[CR10] Carn SA, Krueger AJ, Krotkov NA, Yang K, Evans K (2009). Tracking volcanic sulfur dioxide clouds for aviation hazard mitigation. Nat Hazards.

[CR11] CEMS (2022) Copernicus emergency management service. https://emergency.copernicus.eu/. Accessed 29 June 2022

[CR12] Clark K, Power W, Nishimura Y, Kautoke R’A, Vaiomo’unga R, Pongi ‘A, Fifita M (2011) Characteristics of the 29th September 2009 South Pacific tsunami as observed at Niuatoputapu Island, Tonga. Earth-Sci Rev 107:52–65. 10.1016/j.earscirev.2010.12.001 10.1016/j.earscirev.2011.03.008 PMC4802516 27065478

[CR13] Cummins P, Whatman J, Lahtinen AL, Beavan J, Walace L, Wiens D, Shore P, Heeszel D, Bevis MG, Kendrick E, Taylor FW, Malolo T, Mafi K, Nonu S, Fatai T, Moala ‘A (2007) Interim report on studies of the Mw ~7.9 earthquake of 3 May 2006, Kingdom of Tonga. University of Texas, Institute for Geophysics, Technical Report 204. http://www-udc.ig.utexas.edu/external/facilities/tech_reports/tech_reports/UTIGTR_0204.pdf. Accessed 14 Feb 2022

[CR14] Dickinson WR, Burley DV, Shutler R (1994). Impact of hydro-isostatic holocene sea-level change on the geologic context of Island archaeological sites, Northern Ha’apai group, Kingdom of Tonga. Geoarchaeology.

[CR15] Eliot I, Kumar L, Eliot M et al (2020) Downscaling from whole-island to an island-coast assessment of coastal landform susceptibility to Metocean change in the Pacific Ocean. In: Kumar L (ed) Climate change and impacts in the Pacific. Springer, Cham, pp 225–250. 10.1007/978-3-030-32878-8_5

[CR16] Fakhruddin B, Reinen-Hamill R, Robertson R (2019) Extent and evaluation of vulnerability for disaster risk reduction of urban Nuku’alofa, Tonga. Prog in Disaster Sci 2:100017. 10.1016/j.pdisas.2019.100017

[CR17] Fernando HJS, McCulley JL, Mendis SG, Perera K (2005). Coral poaching worsens tsunami destruction in Sri Lanka. Eos.

[CR18] Ferrario F, Beck MW, Storlazzi CD, Micheli F, Shepard CC, Airoldi L (2014). The effectiveness of coral reefs for coastal hazard risk reduction and adaptation. Nat Commun.

[CR19] Fifita VK, Sanchez AL, Catalan HN, Gordon D (2018) Assessing progress towards the eradication of poverty in the Kingdom of Tonga. Statistics Department Tonga. https://www.poverty.ac.uk/sites/default/files/attachments/Assessing-progress-towards-the-eradication-of-poverty-Tonga-2018.pdf

[CR20] Filizzola C, Lacava T, Marchese F, Pergola N, Scaffidi I, Tramutoli V (2007). Assessing RAT (Robust AVHRR Techniques) performances for volcanic ash cloud detection and monitoring in near real-time: the 2002 eruption of Mt. Etna (Italy). Remote Sens Environ.

[CR21] Finkl CW (2004). Coastal classification: systematic approaches to consider in the development of a comprehensive scheme. J Coast Res.

[CR22] Fritz HM, Borrero JC, Synolakis CE, Okal EA, Weiss R, Titov VV, Jaffe BE, Foteinis S, Lynett PJ, Chan I-C, Liu PL-F (2011). Insights on the 2009 South Pacific tsunami in Samoa and Tonga from field surveys and numerical simulations. Earth Sci Rev.

[CR23] Frohlich C, Hornbach MJ, Taylor FW, Shen C-C, Moala ’A, Morton AE, Kruger J (2009) Huge erratic boulders in Tonga deposited by a prehistoric tsunami. Geology 37:131–134. 10.1130/G25277A.1

[CR24] Garcia-Fry M, Murao O, Bachri S, Moya LA (2022). Land-use microsimulation model for livelihood diversification after the 2010 Merapi volcano eruptions. Transp Res D Transp Environ.

[CR25] GEBCO (2014) GEBCO 2014 grid. https://www.gebco.net/news_and_media/gebco_2014_grid.html. Accessed 2 Feb 2022

[CR26] GFDRR (2022) The January 15, 2022, Hunga Tonga-Hunga Ha'api Eruption and Tsunami, Tonga: Global Rapid Post Disaster Damage Estimation (GRADE) Report. Global Facility for Disaster Reduction and Recovery, Government of Tonga and World Bank. https://reliefweb.int/report/tonga/january-15-2022-hunga-tonga-hunga-haapi-eruption-and-tsunami-tonga-global-rapid-post. Accessed 29 March 2022

[CR27] Government of Tonga (2009a) Tonga: operation Niuatoputapu—day 6, 5 Oct 2009a. Government of Tonga. Published 5 October 2009a. https://reliefweb.int/report/tonga/tonga-operation-niuatoputapu-day-6-5-oct-2009a. Accessed 29 March 2022

[CR28] Government of Tonga (2009b) Tonga: operation Niuatoputapu—day 7, 6 Oct 2009b. Government of Tonga. Published 6 October 2009b. https://reliefweb.int/report/tonga/tonga-operation-niuatoputapu-day-7-6-oct-2009b. Accessed 29 March 2022

[CR29] Government of Tonga (2014) Tonga: tropical cyclone Ian Situation Report No. 3. Government of Tonga. Published 23 January 2014. https://reliefweb.int/report/tonga/tonga-tropical-cyclone-ian-situation-report-no-3-23-january-2014. Accessed 20 Apr 2022

[CR30] Government of Tonga (2022) First official update following the volcanic eruption. Press Release, 18th January, 2022, https://www.gov.to/press-release/first-official-update-following-the-volcanic-eruption/. Accessed 18 Jan 2022

[CR31] GSHHG (2017) Global self-consistent, hierarchical, high-resolution geography database. Version 2.3.7 of June 15, 2017. https://www.ngdc.noaa.gov/mgg/shorelines/. Accessed 18 Jan 2022

[CR32] Gugg G (2022) Ordinary life in the shadow of Vesuvius: surviving the announced catastrophe. In: Świtek B, Abramson A, Swee H (eds) Extraordinary risks, ordinary lives. Springer, Cham, pp 249–275. 10.1007/978-3-030-83962-8_10

[CR33] Gusman AR, Roger J (2022) Hunga Tonga—Hunga Ha’apai volcano-induced sea level oscillations and tsunami simulations. GNS Science webpage. 10.21420/DYKJ-RK41

[CR34] Gusman AR, Roger J, Noble C, Wang X, Power W, Burbidge D (2022). The 2022 Hunga Tonga—Hunga Ha’apai volcano air-wave generated tsunami. Pure Appl Geophys.

[CR35] Hall JD (2004). The South Pacific and southeast Indian Ocean tropical cyclone season 2001–02. Aust Meteorol Mag.

[CR36] Hardy TA, Young IR (1996). Field study of wave attenuation on an offshore coral reef. J Geophys Res Oceans.

[CR37] Harris DL, Rovere A, Casella E, Power H, Canavesio R, Collin A, Pomeroy A, Webster JM, Parravicini V (2018). Coral reef structural complexity provides important coastal protection from waves under rising sea levels. Sci Adv.

[CR38] Havealeta M, Kaho F, Tuipulotu AA (2017) Tonga: cyclone Ian, Ha’apai Islands. In: Bonito S, Minami H (eds) The role of nurses in disaster management in Asia Pacific. Springer, Cham, pp 105–109. 10.1007/978-3-319-41309-9_11

[CR39] Heeszel DS, Wiens DA, Cummins PR, Lahtinen A, Inoue H (2006) The May 3, 2006 (Mw 7.9) Tonga earthquake: shallow thrust or slab tear? Evidence from locally recorded aftershocks. AGU, Fall Meeting 2006, Abstract T21F-05

[CR40] Hunt T, Piper D (2022) Fishers run for lives as Tongan tsunami hits New Zealand, warnings continue. Stuff. https://www.stuff.co.nz/national/127512707/fishers-run-for-lives-as-tongan-tsunami-hits-new-zealand-warnings-continue. Accessed 16 Jan 2022

[CR41] Imamura F, Suppasri A, Arikawa T (2022). Preliminary observations and impact in Japan of the Tsunami caused by the Tonga volcanic eruption on January 15, 2022. Pure Appl Geophys.

[CR42] Japan Times (2022) Japan sees meter-high waves and tsunami warning after massive Tonga eruption. https://www.japantimes.co.jp/news/2022/01/16/national/japan-tsunami-tonga-volcano/. Accessed 16 Jan 2022

[CR43] Jayavanth P, Takai M, Akau’ola S (2009). Disaster and emergency preparedness in Tonga. Southeast Asian J Trop Med Public Health.

[CR44] Jin S, Huang H, Yang Z (2014) Research on Tonga traditional houses adaptive to environment. In: Kao JCM, Sung W-P, Chen R (eds) Green building, materials and civil engineering. CRC Press, pp 891–894. 10.1201/b17568

[CR45] Johnston I (2015). Traditional warning signs of cyclones on remote islands in Fiji and Tonga. Environ Hazards.

[CR46] Karim F, Nandasena NAK (2022) Effectiveness of coral reefs for marine flood reduction. In: 2022 Advances in science and engineering technology international conferences (ASET). IEEE, Dubai, United Arab Emirates, pp 1–4. 10.1109/ASET53988.2022.9734897

[CR47] Kulp S, Strauss BH (2016). Global DEM errors underpredict coastal vulnerability to sea level rise and flooding. Front Earth Sci.

[CR48] Kunkel CM, Hallberg RW, Oppenheimer M (2006). Coral reefs reduce tsunami impact in model simulations. Geophys Res Lett.

[CR49] Lavigne F, Morin J, Wassmer P, Weller O, Kula T, Maea AV, Kelfoun K, Mokadem F, Paris R, Malawani MN, Faral A, Benbakkar M, Saulnier-Copard S, Vidal CM, Tu’I’afitu T, Kitekei’aho F, Trautmann M, Gomez C (2021) Bridging legends and science: field evidence of a large tsunami that affected the Kingdom of Tonga in the 15th century. Front Earth Sci 9:748755. 10.3389/feart.2021.748755

[CR50] Lay T, Ammon CJ, Kanamori H, Rivera L, Koper KD, Hutko AR (2010). The 2009 Samoa-Tonga great earthquake triggered doublet. Nature.

[CR51] LNC (2022) Après une éruption aux Tonga, des mouvements anormaux du niveau de la mer sont à craindre au pays. https://www.lnc.nc/article-direct/faits-divers/mer/societe/nouvelle-caledonie/apres-une-eruption-aux-tonga-des-mouvements-anormaux-du-niveau-de-la-mer-sont-a-craindre-au-pays. Accessed 16 Jan 2022

[CR52] Lolohea SF (2016) Internal Migration in Tonga, 2001–2011: a review of migrant flows and characteristics. MA Thesis (Social Sciences), University of Waikato, Hamilton, New Zealand. https://hdl.handle.net/10289/10741

[CR53] Loriot P, Di Salvo M (2008) Détermination d’un MOS et calcul d’une tache urbaine à partir de la BD TOPO® de l’IGN: étude expérimentale. CERTU. https://hal-lara.archives-ouvertes.fr/hal-02150554

[CR54] Luis JF (2007). Mirone: a multi-purpose tool for exploring grid data. Comput Geosci.

[CR55] Lynett P, McCann M, Zhou Z, Renteria W, Borrero J, Greer D, Fa’anunu O, Bosserelle C, Jaffe B, La Selle S, Ritchie A, Snyder A, Nasr B, Bott J, Graehl N, Synolakis C, Ebrahimi B, Cinar GE (2022) Diverse tsunami genesis triggered by the Hunga Tonga-Hunga Ha’apai eruption. Nature 609:728–733. 10.1038/s41586-022-05170-6 10.1038/s41586-022-05170-6 PMC9472183 35940206

[CR56] Magee AD, Verdon-Kidd DC, Kiem AS, Royle SA (2016). Tropical cyclone perceptions, impacts and adaptation in the Southwest Pacific: an urban perspective from Fiji, Vanuatu and Tonga. Nat Hazards Earth Syst Sci.

[CR57] Meredith ES, Jenkins SF, Hayes JL, Deligne NI, Lallemant D, Patrick M, Neal C (2022). Damage assessment for the 2018 lower East Rift Zone lava flows of Kīlauea volcano. Hawaiʻi Bull Volcanol.

[CR58] Mimura N (1999). Vulnerability of island countries in the South Pacific to sea level rise and climate change. Clim Res.

[CR59] Monismith SG, Rogers JS, Koweek D, Dunbar RB (2015). Frictional wave dissipation on a remarkably rough reef. Geophys Res Lett.

[CR60] Mukul M, Srivastava V, Jade S, Mukul M (2017). Uncertainties in the Shuttle Radar Topography Mission (SRTM) heights: insights from the Indian Himalaya and Peninsula. Sci Rep.

[CR61] Nunn PD, Campbell JR (2020). Rediscovering the past to negotiate the future: How knowledge about settlement history on high tropical Pacific Islands might facilitate future relocations. Environ Dev.

[CR62] Nunn PD, McNamara KE (2019) Failing adaptation in island contexts: the growing need for transformational change. In: Klöck C, Fink M (eds) Dealing with climate change on small islands: towards effective and sustainable adaptation. Göttingen University Press, Göttingen, pp 19–44. 10.17875/gup2019-1210

[CR63] NZ Herald (2022) Volcanic eruption: New Zealand ready to help Tonga; tsunami surge destroys boats, closes beaches in NZ. NZ Herald. https://www.nzherald.co.nz/nz/volcanic-eruption-new-zealand-ready-to-help-tonga-tsunami-surge-destroys-boats-closes-beaches-in-nz/QNI5GQKFSCK52KNHXAT5VAA6DE/. Accessed 16 Jan 2022

[CR64] OCHA (2022a) Daily noon briefing highlights: Tonga. https://www.unocha.org/story/daily-noon-briefing-highlights-tonga. Accessed 19 Jan 2022a

[CR65] OCHA (2022b) Data and Resources. https://data.humdata.org/dataset/cod-ab-ton. Accessed 1 Feb 2022b

[CR66] OCHA (2022c) HOTOSM Tonga Buildings (OpenStreetMap Export). https://data.humdata.org/dataset/hotosm_ton_buildings. Accessed 19 Jan 2022c

[CR67] OCHA (2022d) Kingdom of Tonga: population—Tongatapu, 'Eua, Ha'apai, Vava'u, Niuas (As of 17 January 2022d). https://reliefweb.int/map/tonga/kingdom-tonga-population-tongatapu-eua-haapai-vavau-niuas-17-january-2022d. Accessed 23 Jan 2022d

[CR68] Okal EA, Borrero J, Synolakis CE (2004). The earthquake and tsunami of 1865 November 17: evidence for far-field tsunami hazard from Tonga. Geophys J Int.

[CR69] Okal EA, Fritz HM, Borrero JC (2010) The Samoa tsunami of 29 September 2009: field survey in Tonga and preliminary modeling. In: EGU general assembly conference abstracts, p 7008

[CR70] Pleasance C (2022) Tonga reveals 50 FOOT tsunami destroyed ALL houses on one island while just two are left on another in 'unprecedented disaster' as death toll climbs to three and new pictures reveal extent of devastation. Mailonline and Reuters. https://www.dailymail.co.uk/news/article-10413485/Tonga-eruption-Aid-planes-land-island-damage.html. Accessed 19 Jan 2022

[CR71] Reardon GF, Oliver J (1983). The impact of cyclone Isaac on buildings on Tonga. J Wind Eng Ind Aerodyn.

[CR72] Roger J, Dudon B, Krien Y, Zahibo N (2014) Discussion about tsunami interaction with fringing coral reef. In: Kontar YA, Santiago-Fandiño V, Takahashi T (eds) Tsunami events and lessons learned. Springer, Dordrecht, pp 161–176. 10.1007/978-94-007-7269-4_8

[CR73] Rogers G (1981). The evacuation of Niuafo’ ou, an outlier in the kingdom of Tonga∗. J Pac Hist.

[CR74] Sattler DN, Lousi U, Graham JM, Latu V, Johnson J, Helu SL (2020) Climate change in Tonga: risk perception and behavioral adaptation. In: Leal Filho W (ed) Managing climate change adaptation in the Pacific region. Springer, Cham, pp 273–292. 10.1007/978-3-030-40552-6_14

[CR75] Scott DA, Browne C (1989) Tonga. In: Scott DA, Browne C (eds) Economic development in seven Pacific Island countries. International Monetary Fund, pp 135–156. 10.5089/9781557750358.071

[CR76] Searcy C, Dean K, Stringer W (1998). PUFF: a high-resolution volcanic ash tracking model. J Volcanol Geotherm Res.

[CR77] Simpson A, Johnson RW, Cummins P (2011). Volcanic threat in developing countries of the Asia-Pacific region: probabilistic hazard assessment, population risks, and information gaps. Nat Hazards.

[CR78] SRTM (2015) NASA Shuttle Radar Topography Mission (SRTM) Version 3.0 Global 1 arc second data released over Asia and Australia. https://www.earthdata.nasa.gov/news/nasa-shuttle-radar-topography-mission-srtm-version-3-0-global-1-arc-second-data-released-over-asia-and-australia. Accessed 26 Jan 2022

[CR79] Sykes WR (1981) The vegetation of late, Tonga. Allertonia, pp 323–353

[CR80] Tang L, Titov VV, Wei Y, Mofjeld HO, Spillane M, Arcas D, Bernard EN, Chamberlin C, Gica E, Newman J (2008). Tsunami forecast analysis for the May 2006 Tonga tsunami. J Geophys Res.

[CR81] Taylor PW (1991) The geology and petrology of Niuafo’ou Island, Tonga: subaerial volcanism in an active back-arc basin. Macquarie University. M.Sc. Thesis. 10.25949/19440851.V1

[CR82] Terry JP, Goff J, Winspear N, Bongolan VP, Fisher S (2022). Tonga volcanic eruption and tsunami, January 2022: globally the most significant opportunity to observe an explosive and tsunamigenic submarine eruption since AD 1883 Krakatau. Geosci Lett.

[CR83] Thomas BEO, Roger J, Gunnell Y, Sabinot C, Aucan J (2021). A low-cost toolbox for high-resolution vulnerability and hazard-perception mapping in view of tsunami risk mitigation: application to New Caledonia. Int J Disaster Risk Reduct.

[CR84] TSD (2017) Tonga 2016 Census of population and housing Volume 1 Basic tables and administrative reports Second edition. Tonga Statistics Department, https://tongastats.gov.to/census/population-statistics/#22-60-wpfd-2016-1498707296. Accessed 23 Jan 2022

[CR85] TSD (2019) Tonga Census 2016 Census of population and housing Volume 2 Analytical report. Tonga Statistics Department, https://tongastats.gov.to/census/population-statistics/#22-60-wpfd-2016-1498707296. Accessed 23 Jan 2022

[CR87] UNISDR (2005) Hyogo framework for action 2005–2015: building the resilience of nations and communities to disasters

[CR88] UNISDR (2009) Reducing disaster risks through science: issues and actions

[CR89] UNOSAT (2022) 15 January 2022 volcanic eruption and tsunami. Preliminary satellite-based comprehensive damage assessment report. Tongatapu, Eua, and Ha’apai divisions of the Kingdom of Tonga. United Nations Satellite Centre (UNOSAT) report. https://reliefweb.int/sites/reliefweb.int/files/resources/UNOSAT_ComprehensiveDamageAssessment_Tonga_20220204.pdf. Accessed 23 Jan 2022

[CR90] UNOSAT Maps (2022) Damage assessment in Tonga islands as of 20 January 2022. https://experience.arcgis.com/experience/5be5d28e6fe740ae9ea8d940f6ef112b/?draft=true. Accessed 23 Jan 2022

[CR91] USGS HDDS (2022) USGS Hazards Data Distribution System. https://hddsexplorer.usgs.gov/. Accessed 29 June 2022

[CR92] Vainikolo LC (2021) Small and Isolated Vava’u, Tonga: from weakness to strength during COVID-19? In: Campbell Y, Connell J (eds) COVID in the Islands: a comparative perspective on the Caribbean and the Pacific. Springer, Singapore, pp 207–218. 10.1007/978-981-16-5285-1_11

[CR93] Webley P, Mastin L (2009). Improved prediction and tracking of volcanic ash clouds. J Volcanol Geotherm.

[CR94] Wessel P, Luis JF, Uieda L, Scharroo R, Wobbe F, Smith WHF, Tian D (2019). The Generic Mapping Tools version 6. Geochem Geophys Geosyst.

[CR95] WHO (2009) Tonga: tsunami situation report 1. World Health Organization. Published 2 October 2009. https://reliefweb.int/report/tonga/tonga-tsunami-situation-report-1. Accessed 29 March 2022

[CR96] Wilson KJ, Power WL, Nishimura Y, ‘Atelea Kautoke R, Vaiomo’unga R, Mori H, Pongi ‘A, Fifita M, Vaoahi M, Teukava S (2009) Post-tsunami survey of Niuatoputapu Island, Tonga, following the 30th September 2009, South Pacific tsunami, GNS Science Report 2009/71. http://itic.ioc-unesco.org/images/docs/SR-2009-71.pdf. Accessed 29 March 2022

[CR97] World Bank (2022) Rebuilding tsunami-affected homes in remote islands of Tonga. The World Bank. Published 10 April 2014. https://www.worldbank.org/en/results/2014/04/10/rebuilding-tsunami-affected-homes-in-remote-islands-of-tonga. Accessed 29 March 2022

[CR98] World Risk Report (2018) https://weltrisikobericht.de/wp-content/uploads/2019/03/190318_WRR_2018_EN_RZonline_1.pdf. Accessed 13 Feb 2022

