# Supplementary material for: A method for evaluating population and infrastructure exposed to natural hazards: tests and results for two recent Tonga tsunamis
Source: Geoenvironmental Disasters. 2023 Feb 16;10(1):4. doi: 10.1186/s40677-023-00235-8 (PMC9934511; doi:10.1186/s40677-023-00235-8)
Supplement: Supplementary file 1 — Additional file 1. Shapefiles of the populated areas in Tong, all details are provided in the associated Word document (Supplementary Material Dataset PA.docx). [file 40677_2023_235_MOESM1_ESM.zip › supp_material/Supplementary Material Dataset PA.docx]

**Supplementary Material Dataset PA**

The dataset contains:

- Shapefile of the populated areas (*pop_area.shp*);
- Shapefile of the populated areas undefined (*pop_area_nd.shp*);
- Shapefiles of the coastal populated areas for each elevation band up to +30 m (*pop_area_under01m.shp, pop_area_under05m.shp, pop_area_under10m.shp, pop_area_under15m.shp, pop_area_under20m.shp, pop_area_under25m.shp, pop_area_under30m.shp*);
- Shapefiles of the coastal populated areas undefined for each elevation band up to +30 m (*pop_area_under01m_nd.shp, pop_area_under05m_nd.shp, pop_area_under10m_nd.shp, pop_area_under15m_nd.shp, pop_area_under20m_nd.shp, pop_area_under25m_nd.shp, pop_area_under30m_nd.shp*);

Legend for the populated area shapefiles

| code3 | Code of the village |
| --- | --- |
| name3 | Postal code of the village |
| _ADM3_NAME | Name of the village |
| _ADM2_PCOD | Code of the district |
| _ADM2_NAME | Name of the district |
| _ADM1_PCOD | Code of the division |
| _ADM1_NAME | Name of the division |
| _pop_2016 | Population in the village in 2016 |
| a_pa | Area of the whole populated area in the village |
| a_pas | Area of the populated area of interest |
| pop_pasF | Population in the populated area of interest |
| a_pasc | Area of the coastal populated area |
| pop_pascF | Population in the coastal populated area |

Legend for the populated area undefined shapefiles

| ADM3_NAME | Name of the undefined populated area |
| --- | --- |
| households | Number of buildings |
| pop_calcul | Calculation of the number of people on the basis of 5.5 individuals/household |
| a_pa | Area of the whole populated area in the village |
| a_pas | Area of the populated area of interest |
| pop_pasF | Population in the populated area of interest |
| a_pasc | Area of the coastal populated area |
| pop_pascF | Population in the coastal populated area |
